# Supplementary material for: Intraparietal stimulation disrupts negative distractor effects in human multi-alternative decision-making
Source: eLife. 2023 Feb 22;12:e75007. doi: 10.7554/eLife.75007 (PMC9946441; doi:10.7554/eLife.75007)
Supplement: Transparent reporting form [file elife-75007-transrepform1.docx]

***eLife’s* transparent reporting form**

We encourage authors to provide detailed information *within their submission* to facilitate the interpretation and replication of experiments. Authors can upload supporting documentation to indicate the use of appropriate reporting guidelines for health-related research (see [EQUATOR Network](http://www.equator-network.org/%20)), life science research (see the [BioSharing Information Resource](https://biosharing.org/)), or the [ARRIVE guidelines](http://www.plosbiology.org/article/info:doi/10.1371/journal.pbio.1000412) for reporting work involving animal research. Where applicable, authors should refer to any relevant reporting standards documents in this form.

If you have any questions, please consult our Journal Policies and/or contact us: [editorial@elifesciences.org](mailto:editorial@elifesciences.org).

**Sample-size estimation**

- You should state whether an appropriate sample size was computed when the study was being designed
- You should state the statistical method of sample size computation and any required assumptions
- If no explicit power analysis was used, you should describe how you decided what sample (replicate) size (number) to use

Please outline where this information can be found within the submission (e.g., sections or figure legends), or explain why this information doesn’t apply to your submission:

This study adopted a sample size based on those in previous studies that involved applying rTMS to the intraparietal region. These studies that showed behavioural effects involved between 10 to 15 participants. The current study involved a sample size of 31 that surpassed this range. This is explained in the section - *Methods, Participants* (Page 35).

**Replicates**

- You should report how often each experiment was performed
- You should include a definition of biological versus technical replication
- The data obtained should be provided and sufficient information should be provided to indicate the number of independent biological and/or technical replicates
- If you encountered any outliers, you should describe how these were handled
- Criteria for exclusion/inclusion of data should be clearly stated
- High-throughput sequence data should be uploaded before submission, with a private link for reviewers provided (these are available from both GEO and ArrayExpress)

Please outline where this information can be found within the submission (e.g., sections or figure legends), or explain why this information doesn’t apply to your submission:

This study required participants to complete a multi-alternative value-based decision-making task similar to that of previous studies. This is detailed in the section - *Methods, Decision-Making Task* (Pages 36-38).

The quality of the eye movement data was estimated moment-by-moment using an in-built algorithm of the Tobii TX300 eye tracker. The inclusion/exclusion criteria of the eye movement data were described in the sections - *Methods, Eye movement recording and preprocessing* (Page 41, Lines 732-743) and *Methods, Statistical Analysis, Eye-tracking Data Analysis* (Pages 44-45). Specifically, we were unable to identify sufficient gaze shifts in five participants, which were excluded from the eye-tracking data analysis.

**Statistical reporting**

- Statistical analysis methods should be described and justified
- Raw data should be presented in figures whenever informative to do so (typically when N per group is less than 10)
- For each experiment, you should identify the statistical tests used, exact values of N, definitions of center, methods of multiple test correction, and dispersion and precision measures (e.g., mean, median, SD, SEM, confidence intervals; and, for the major substantive results, a measure of effect size (e.g., Pearson's r, Cohen's d)
- Report exact p-values wherever possible alongside the summary statistics and 95% confidence intervals. These should be reported for all key questions and not only when the p-value is less than 0.05.

Please outline where this information can be found within the submission (e.g., sections or figure legends), or explain why this information doesn’t apply to your submission:

Explanations of the statistical analysis methods are provided in *Methods, Statistical Analysis* (Pages 41-45) and also repeated in various sections of *Results* (Pages 7-27).

The sample sizes of the current study is 31. Error bars indicating standard errors were presented in all bar charts.

The statistical test used and the sample size of each test (in form of degree-of-freedom) were described in both the *Results* and *Methods* sections. The mean and standard error of the key results can be found in the figures in form of bar charts.

The exact p values of each test were reported to 3 decimal places in the *Results* section.

(For large datasets, or papers with a very large number of statistical tests, you may upload a single table file with tests, Ns, etc., with reference to sections in the manuscript.)

**Group allocation**

- Indicate how samples were allocated into experimental groups (in the case of clinical studies, please specify allocation to treatment method); if randomization was used, please also state if restricted randomization was applied
- Indicate if masking was used during group allocation, data collection and/or data analysis

Please outline where this information can be found within the submission (e.g., sections or figure legends), or explain why this information doesn’t apply to your submission:

Not applicable, since the current study involved a within-subject design with no group allocation. For each participant, the order of the rTMS stimulation site (i.e., MIP/MT) was randomly assigned.

**Additional data files (“source data”)**

- We encourage you to upload relevant additional data files, such as numerical data that are represented as a graph in a figure, or as a summary table
- Where provided, these should be in the most useful format, and they can be uploaded as “Source data” files linked to a main figure or table
- Include model definition files including the full list of parameters used
- Include code used for data analysis (e.g., R, MatLab)
- Avoid stating that data files are “available upon request”

Please indicate the figures or tables for which source data files have been provided:

The behavioural data, eye-tracking data, anonymized MRI grey matter volume data collected and analysed during the current study are available on Dryad: <https://doi.org/10.5061/dryad.ngf1vhhzn>.
